# General Practitioners' preferences and use of educational media: a German perspective

**DOI:** 10.1186/1472-6963-9-31

**Published:** 2009-02-16

**Authors:** Horst Christian Vollmar, Monika A Rieger, Martin E Butzlaff, Thomas Ostermann

**Affiliations:** 1 Institute of General Practice and Family Medicine, Witten/Herdecke University, Alfred-Herrhausen-Str. 50, 58448 Witten, Germany; 2 Fraunhofer Institute for Systems and Innovation Research (ISI), Karlsruhe, Breslauer Str. 48, 76139 Karlsruhe, Germany; 3 Institute of Occupational andSocial Medicine, University of Tübingen, Tübingen, Germany; 4 Faculty of Medicine, Witten/Herdecke University, Witten, Germany; 5 Chair of Medical Theory and Complementary Medicine, Witten/Herdecke University, Witten, Germany

## Abstract

**Background:**

Several studies suggest that General Practitioners (GPs) prefer "traditional" media such as journals or quality circles when they are seeking out different options to meet their continuing medical education (CME) requirements. A survey was designed in order to gain a better understanding of German General Practitioners' preferences for different forms of educational media that will meet their CME needs.

**Methods:**

Four hundred and forty nine (N = 449) German physicians were contacted to take part in this study on the occasion of one of their quality circle meetings. The participating physicians received a standardized 26-item-questionnaire that surveyed their preferences for different forms of educational media. A factor analysis was performed in order to determine whether the observed variables can be explained largely or entirely in terms of the underlying patterns.

**Results:**

Two hundred and sixty-four physicians with an average age of 51.1 years participated (28.5% female, 71.5% male). We found that GPs favor learning environments such as: *journals*, *colleagues*, and *quality circles*. New media like the *internet *was used less often for their learning activities, even though the usage of the internet in general was quite high. The most important requirements for media in medical education as perceived by the participants were its *relevancy for daily practice *and *dependability*.

**Conclusion:**

Despite a growing use of the Internet it seems that German GPs favor "classical/traditional" settings for their learning activities. These results should be taken into consideration when planning CME or CPD programs or other learning activities.

**Trial registration:**

Current Controlled Trials ISRCTN36550981.

## Background

In the context of medical care recent, significant developments in the areas of technology, therapy and new forms of medical treatment ask for a timely diffusion of available evidence on interventions. Thus the translation of knowledge into practice is essential in order to maintain and increase the quality of daily practice [1]. As a consequence, health professionals such as General Practitioners (GPs) have to ensure that their knowledge and skills are up to date. Since the early Eighties, a number of studies and reviews have demonstrated the positive influence continuing medical education (CME) has on a physicians' knowledge and competence [2-4]. Over the same period of time, peer review groups and quality circles (QCs) have formed, i.e. regional meetings of GPs including specialists in order to discuss both clinical topics and new developments in politics and funding. The first QCs of GPs in Germany were founded in 1991/92 to help GPs tackle problems of quality management in the patients' daily care. Today, the participation of German GPs in QCs is mandatory in order to be part of most of the governmental funded disease management programs or to be part of model projects and initiatives from health insurers. Hence, nearly 50% of all German GPs are now organized in QCs [5]. Parallel to this process, CME was rendered mandatory for GPs in Germany in January 2004, which is still well behind other international trends where a recognizable shift from "continuing medical education" (CME) to "continuing professional development" (CPD) has been observed [6-8]. In CME/CPD, the application of new information technologies is recommended in order to have a lasting impact on the physicians' working environment and their learning behaviors [9-11].

The design of a unified strategy to apply the relatively new tools of e-learning in medicine has proved to be a challenge for both users and content creators [12]. To optimize the choices involved, it is necessary to survey the physicians, their needs and to identify the underlying learning types and preferences of GPs in daily practice [9,13].

To identify relevant publications on this topic, we carried out searches on Medline and other Internet research sites, using the terms "media", "preferences", and "physicians". We found a few articles which give relevant information about the (learning media) preferences of physicians respectively GPs. Table 1 provides an overview of the methodologies and key results of these articles. Data from older surveys showed that family physicians indicated colleagues most often as information sources, followed by journals and books [14,15]. This outcome corresponded with results in other professions [14].

**Table 1:** Surveys of CME preferences

**Study Source**	Sampling method*	**Sample**	**Favorite learning formats (or offers)**	**Country**
Slotnick et al. 1994 [57]	R	Practicing physicians from the AMA Physician master file	Other research questions	US
Haug 1997 [15]	6 × R 6 × C	Meta-analytic study of 12 studies with 20 strata groups, mostly General Practitioners and Family Physicians	Books, journals, colleagues, courses, and meetings (less: library references)	US
Hayward et al. 1997 [58]	R	Physicians	Other research questions	Canada
Tinsley et al. 1998 [59]	R	Psychiatric Physicians and Family Physicians	Other research questions	US (Minnesota)
Verhoeven et al. 1999 [38]	R	General Practitioners	Drug reference and private books, colleagues (less CD-Rom and internet)	Netherlands
Smith et al. 2000 [60]	R	14 medical societies with antimicrobial-resistance educational offerings	All societies supported educational offerings, most frequently as professional meetings, followed by audiotapes, computer programs, Internet sites, or print-based self-study materials.	US
Brown et al. 2001 [29]	R	Members of the Society for Healthcare Epidemiology of America	Journal articles, local ground rounds and meetings	US
Butzlaff et al. 2001 [61]	C	Physicians in hospitals	Colleagues, journals, books, conferences (less: internet, consultants, and pharmaceutical representatives)	Germany
Slotnick et al. 2001 [62]	R	Physicians (sampled from doctors with faculty appointments and no such appointments)	Colleagues, journals, review articles	US (North Dakota)
Stancic et al. 2003 [63]	C	Physicians of 4 rural areas	Live lectures out of the offering of the three formats live lectures, videotapes, and World Wide Web-based training	US (Texas)
Sargeant et al. 2004 [41]	P	Physicians of three Canadian universities	Other research questions	Canada
Bower et al. 2008 [39]	R	Physicians	Other research questions	US (Oregon)

Butzlaff et al. 2002 [16] Vollmar et al. 2008 [17]	C	General Practitioners after initial and after a period of 6 years	Journals, colleagues and quality circles (relative constant)	Germany

Sampling method: R = Random, C = Convenience, P = Purposive (for focus groups)

With respect to the German health care system answers to these questions were initially obtained by a pilot-survey with 72 GPs conducted in 2001 [16]. Results indicated that GPs in Germany at that time preferred traditional learning environments like "journals", "colleagues" and "quality circles". Only 21% of the participating GPs used the internet for educational activities, which was less than their use for consulting pharmaceutical representatives (28%). Six years later their preferences remained relatively stable [17].

While the usage of the internet by ambulatory care physicians has increased from 54*% *(2001) to 82*% *(2007), it still is unclear if the preferred method of learning has also changed [18]. Thus, to study the primary care physicians' actual preferences for different educational media, we conducted a survey of German office-based general practitioners with a larger sample size, which allowed us to gather more information about the utilization, efficacy and suggested requirements for different forms of educational media. Additionally, we carried out a factor analysis of the survey items in order to get a better understanding of the different learning types and behavioral patterns of the physicians who participated in this study.

## Methods

### Participants

**Figure 1:**
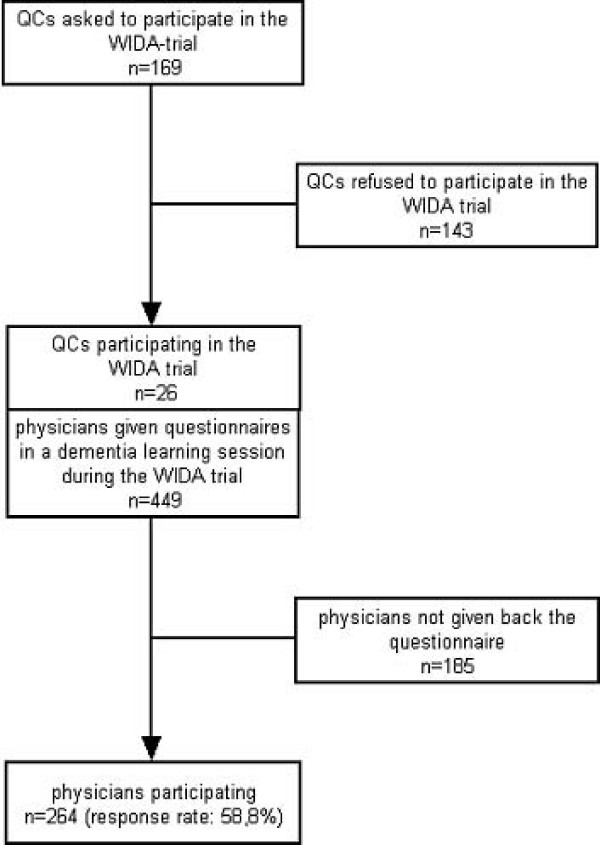
**Response and return rates of GPs**.

Survey data for this study were collected as part of the WIDA-trial (registered in Current Controlled Trials [ISRCTN36550981]). WIDA is the German acronym for "knowledge transfer for dementia in General Practice" [19]. To get a sufficient number of participants for the cluster-randomized WIDA-trial, a total of 169 quality circles (QCs) originally were asked to take part in this study (Figure 1). Of those, 26 QCs agreed to participate and the GPs in the QCs were randomly assigned to one of the two methods. At the first meeting of the WIDA-study, where GPs were asked for their informed consent, all physicians were asked to fill out a questionnaire about learning media preferences (including the GPs who refused to participate in the WIDA-trial). GPs who had previously agreed to take part in our study were handed out a standardized 26-item-questionnaire (N = 449). Two hundred and sixty-four physicians completed the questionnaire (response rate: 58.8%, Figure 1). The questionnaire covered the following topics (see Table 2 and Table 3 for a complete item list):

**Table 2:** Structure and results of the questionnaire – Part I

***How often do you ****use**** the following educational media?/How would you assess the ****efficiency ****(ratio of time spent on information gain)?***		**Degree of utilization/Assessment of efficiency**			**Correlation between utilization and efficiency**
			
		Never/little	Sometimes/medium	Often/high	
**Colleagues**	Use of:	3 (1.1%)	104 (39.8%)	154 (59%)	0.304*
	Efficiency:	20 (7.8%)	81 (31.4%)	157 (60.9%)	
**Quality circles**	Use of:	2 (0.8%)	62 (23.6%)	199 (75.7%)	0.468*
	Efficiency:	8 (3.1%)	90 (35%)	159 (61.9%)	
**Conferences/Congresses**	Use of:	10 (3.8%)	151 (57.6%)	101 (38.5%)	0.343*
	Efficiency:	25 (9.8%)	116 (45.5%)	114 (44.7%)	
**Books**	Use of:	7 (2.7%)	151 (57.6%)	104 (39.7%)	0.326*
	Efficiency:	12 (4.7%)	117 (45.5%)	128 (49.8%)	
**Pharmaceutical Representative**	Use of:	61 (23.4%)	160 (61.3%)	40 (15.3%)	0.451*
	Efficiency:	187 (73.6%)	61 (24%)	6 (2.4%)	
**University Representative**	Use of:	91 (38.4%)	130 (54.9%)	16 (6.8%)	0.579*
	Efficiency:	92 (36.7%)	104 (41.4%)	55 (21.9%)	
**Scientific Journals**	Use of:	2 (0.8%)	91 (35.1%)	166 (64.1%)	0.408*
	Efficiency:	16 (6.3%)	111 (43.4%)	129 (50.4%)	
**Internet**	Use of:	83 (32%)	123 (47.5%)	53 (20.5%)	0.512*
	Efficiency:	90 (37.2%)	87 (36%)	65 (26.9%)	

*all correlations are significant on a level of 0.01 (two-sided)

**Table 3:** Structure and results of the questionnaire – Part II

**How important do you rate the following requirements in medical information media?**	**Personal weighting**		
	
	unimportant	less important	very important
Fast	13 (5.2%)	91 (36.4%)	146 (58.4%)
Reliable/scientific	0	23 (9.1%)	229 (90.9%)
Concise	0	71 (28.2%)	181 (71.8%)
Relevant to practice	0	17 (6.7%)	236 (93.3%)
With graphical material	59 (23.2%)	151 (59.4%)	44 (17.3%)
Problem-based	30 (11.9%)	145 (57.3%)	78 (30.8%)
German language	25 (9.9%)	125 (49.4%)	103 (40.7%)
Interactive	79 (31.1%)	137 (53.9%)	38 (15%)
User friendly	8 (3.2%)	72 (28.7%)	171 (68.1%)
Cost-effective	30 (12%)	130 (51.8%)	91 (36.3%)

• Utilization of educational media (8 items, using a 3-point ordinal scale with the scoring options: 0 = "never", 1 = "sometimes", and 2 = "often")

• Efficacy of educational media (8 items, using a 3-point ordinal scale with the scoring options: 0 = "little", 1 = "medium", and 2 = "high")

• Requirements for educational media (10 items using a 3-point ordinal scale with the scoring options: 0 = "unimportant", 1 = "less important", and 2 = "very important").

The questionnaire was completed with general items on socio-medical demographic information such as age, gender, year of examination, internet usage.

### Statistical analysis

As mentioned, we used two levels of statistical analysis in this study: (1) a micro, or item-wise level concentrating on the exploratory analysis for the items concerned with the use of educational media and (2) a macro level to discover patterns within the relationships among the items. Both macro- and micro-level analysis are used to accomplish the objective of obtaining valid information on the utilization and perceived requirements for educational media. For the macro level we performed a factor analysis on the relevant items using a principal component analysis with a varimax rotation, to determine whether the observed variables can be explained largely or entirely in terms of underlying patterns. Additionally, Cronbach's alpha was calculated as an indicator of the internal consistency of the scales. Finally, item values were summed and then transformed into a 0–100 scale, where higher scores indicated greater levels of agreement. To classify the GPs into different learning types we assigned them to the category where they scored highest.

Statistical analysis of the sample for both levels included descriptive statistics giving item percentages or subscale means with confidence intervals of 95%. To determine the correlation between the subscales, we calculated the Pearson's correlation coefficient.

Due to the exploratory character of the study dichotomous subgroups of survey participants were tested using the Mann-Whitney-U-Test for independent samples for continuous variables (i.e. subscales). We judged p < 0.05 to be significant, and p > 0.05 – 0.10 as a trend. Data analysis was conducted using SPSS Version 15.01.

## Results

Of the 264 respondents, 28.5% were female and 71.5% were male. The average age of the sample was 51.1 (± 7.1) years, ranging in age from 30 to 68 years; 154 of the physicians were GPs, 76 were internists mainly working in primary care and 12 physicians were in other specialties. A comprehensive overview of demographic data for the participating physicians is provided in Table 4.

**Table 4:** Demographic data of participating physicians

	**n (%)**	**n (%)**	**All**	**n (missing)**
Sex	Female 71 (28.5)	Male 178 (71.5)	249	249 (15)
General Practitioner	50 (32.5%)	104 (67.5%)	154	242 (22)
Internist	15 (19.7%)	61 (80.3%)	76	
Other Specialization	6 (50%)	6 (50%)	12	
				
Working in single practice	23 (19.8%)	93 (80.2%)	116	248 (16)
Working in group practice	47 (38.2%)	76 (61.8%)	123	
Hospital, polyclinic or other institution	1 (11.1%)	8 (88.8%)	9	
				
No internet access	4 (36.4%)	7 (63.6%)	11	248 (16)
Internet access at practice	6 (54.5%)	5 (45.5%)	11	
Internet access at home	33 (28%)	85 (72%)	118	
Internet access at practice and home	28 (25.9%)	80 (74.1%)	108	
				
Internet use less than once week	21 (33.3%)	42 (66.7%)	63	244 (20)
Internet use 1–3 times a week	26 (35.6%)	47 (64.4%)	73	
Internet use 4–6 times a week	6 (18.7%)	26 (81.3%)	32	
Internet use daily	16 (21.1%)	60 (78.9%)	76	
				
	**Years (SD)**			**n (missing)**
Average age (SD) Minimum-Maximum	49.7 (7.3) 33–66	51.7 (7) 30–68	51.1 (± 7.1) 30–68	250 (14)
				
	**Year (SD)**			
Year of last examination at university	1984 (± 7.8)	1982 (± 7.3)	1982.8 (± 7.4)	242 (22)

### Internet access and utilization

Eleven of the participating physicians (4.4%) had no internet access; all of the others had internet access at their practice, their home or both (Table 4). Sixty three physicians (25.8%) used the internet less than once a week, while 74 (31.1%) used it on a daily basis (Table 4).

### Utilization and efficacy

For their learning activities the general practitioners most frequently used quality circles (75.6%), followed by journals (64.1%), and then colleagues (58.8%). These educational media were also valued with regard to their learning-efficacy. The internet, pharmaceutical representatives, and the university representatives were used less and were also less valued than other approaches to meet their learning needs. There were significant correlations between utilization and efficacy for every item (p < 0.01, Table 2).

### Requirements

The most favored attributes regarding tools for continuing medical education were "relevant to daily practice" (very important: 92.5%), "reliable" (89.8%), and "concise" (71%). The less favored ones were "cost-effective" (36.1%), "problem-based" (30.8%), "graphical material" (17.3%), and "interactive" (15%) (Table 3), which are related to the key attributes of web based learning.

### Factor analysis

When analyzing the utilization of learning media, we found a stable 3-factor-model. The cumulative variance explained by this model was 48.7%. With a value of Cronbach's alpha of 0.6 the internal consistency of the 8-item pool can be regarded as acceptable for descriptive purposes [20]. The resulting factors describe three different types of users: The first factor with two items comprising the items *internet *and *books *describes the more *intrinsic *motivated user, who was learning on his/her own. Factor 2 consists of the 3 items *journals *(r = 0.66), *quality circles *(r = 0.63), and *colleagues *(r = 0.61). The items for this factor are more equal in respect to their factorial values and according to Wolf et al. (1986) they can be described as *collegial interaction *[21]. The remaining items lead to factor 3 which is polar to the first factor. It describes the more *extrinsic *oriented physician who likes to meet *university representatives *and *pharmaceutical representatives *and also joins in at *conferences*. Correlational analysis of the scales did not yield relationships between them, showing the factors were independent (Table 5).

**Table 5:** Factor analysis of utilization items

	**Resulting scales with factor loadings**			**Item Mean (SD)**
**Items on utilization of educational resources**	Intrinsic motivation	Collegial & interactive	Extrinsic motivation	(Range: 0–2)
1. Internet	**0.74**			0.9 (0.8)
2. Books	**0.51**			1.4 (0.6)
3. Journals	0.39	**0.66**		1.6 (0.5)
4. Quality Circles	-0.36	**0.63**		1.8 (0.5)
5. Colleagues		**0.61**		1.6 (0.6)
6. University representatives			**0.68**	0.6 (0.6)
7. Conferences	0.31		**0.62**	1.4 (0.6)
8. Pharmaceutical representatives	0.32		**0.59**	0.9 (0.7)

Factor loadings > 0.5 are marked bold

A second factor analysis was carried out for the items dealing with requirements on educational media (Table 6). The cumulative variance explained by this model was 49.4%. Again, with a value of Cronbach's alpha value of 0.6, the internal consistency of the 10-item pool was sufficient [20].

**Table 6:** Factor analysis of requirement items

	**Resulting scales with factor loadings**			**Item Mean (SD)**
**Items on requirements of education media**	Didactic quality	Suitability	Efficacy	(Range: 0–2)
1. Problem based	**0.74**			1.2 (0.6)
2. Interactive	**0.66**			0.8 (0.6)
3. Graphical Material	**0.63**			0.9 (0.6)
4. German language	**0.55**			1.3 (0.6)
5. Relevant to practice		**0.81**		1.9 (0.3)
6. Reliable		**0.71**		1.9 (0.3)
7. Concise		**0.60**	0.30	1.7 (0.5)
8. Fast			**0.76**	1.5 (0.6)
9. Cost-effective			**0.68**	1.3 (0.6)
10. User friendly*	0.40		0.41	1.7 (0.5)

Factor loadings > 0.5 are marked bold/*has no sufficient loading (see text)

The first factor derived in this analysis comprised the following four items: *problem based interactive*, *graphical material*, and *German language*. All of them describe the *didactic quality *of the educational media and thus the factor is named accordingly. The second factor summarizes the items *relevant to practice*, *reliable*, and *concise *which comprise the *suitability *of educational media in daily practice. The last factor describes the *efficacy *of educational media and consists of the two items *fast *and *cost-effective*. Correlation analysis between the scales showed a clear independence of the factors (correlation coefficient r between -0.09 and 0.251). Although some of the items showed differences within age and internet usage in particular, the Mann-Whitney-U-Test did not find any significant changes in the subscale median with respect to the socio-demographic parameters such as gender, type of practice, internet access, and internet usage. The factor *suitability *contained the items which were also the highest ranked ones, resulting in subscale means greater than 90.0 for all of the parameters. The other two factors showed a greater variability within the social demographic subgroups of our sample. Regarding *didactic quality *we found high values in physicians who used the internet less than once a week (scale value 59.0) whereas the lowest values were obtained from physicians using the internet quite frequently (scale value 45.6 from those physicians who used the internet 4–6 times a week). In the same subgroup however the efficacy was valued in a proportionally opposite manner to the didactic quality: those participants who used the internet quite often also had higher demands on efficacy of learning media (mean scale values 72.1 and 75.8) than those who did not use the internet that often (mean scale value 65.0). Efficacy was ranked highest with the small group of physicians working in a hospital based environment (mean scale value 81.3).

## Discussion

Learning media preferences of GPs are an important topic within the research agenda on knowledge translation. Our descriptive survey provides a comprehensive analysis of the GPs preferences for educational media and we could predict that German general practitioners in general favor the "classical" learning environments such as: *journals*, *colleagues*, and *quality circles*. The so called new media like the *internet *were less used and less valued for educational activities, even though the usage of the internet in general was quite high. This finding is supported by other studies [18,22].

With respect to the GPs' preferences regarding utilization, the factor analysis was able to identify three different types of learners: the *intrinsic*, the *extrinsic *and the *collegial or interactive *learner. The first two types have already been described in other learning environments such as second language learning [23,24]. The third type *collegial and interactive *was described for the first time in the work of Wolf and colleagues [21]. Our data suggests that about 70% of the physicians want to exchange ideas and discuss actual trends with colleagues *collegial and interactive *rather than to meet experts *extrinsic *or to read a book *intrinsic*. An explanation for this high fraction might be that our survey was conducted with GPs participating in quality circles, where collegial interaction is a desirable and quite common characteristic.

For the GPs preferences regarding their media requirements, the factor analysis revealed three "factors": *didactic quality*, *suitability*, and *efficacy*. For those physicians who used the Internet frequently *efficacy *was most important, whereas for GPs who used the Internet only marginally, *didactic quality *was more relevant. Due to the descriptive nature of the factor analysis further studies are necessary in order to confirm the discovered types and to explore this field in more detail. Also, the three point scale used in this study could be hypothesized to be too short to optimize the variation [25]. This might be the reason why factor analyses resulted in poor solutions of less than 50% of the total variance. Future work with this instrument should improve the scale's psychometric properties i.e. by using a 5-point Likert scale or a truly continuous scale.

### Limitations

Considering a possible setting bias, it has to be mentioned that data were collected during a QC session where GPs were asked to participate both in a clinical trial on dementia (the WIDA-trial) and – independently – in this survey [19]. This may explain the relative positive appraisal for the usage and efficacy of quality circles. Having in mind that every second German GP is organized in QCs, the potential for a serious bias is within reasonable limits. Anyhow, it has to be taken into account that of 169 QCs, only 26 (15%) agreed to participate and only 264 GPs out of 449 responded. Even with a slightly older study population and a lower percentage of women than the GP population as a whole, there is still a considerable agreement with the available demographic normative data regarding office-based physicians in Germany [26].

We used a convenience sampling method which means that every GP during a QC session of the WIDA-trial were asked to fill out the questionnaire for this survey. This setting was chosen to reach a large number of GPs, considering the fact that the response rate in postal questionnaires is often unsatisfactory [27].

Again, these results are comparable to samples in other studies [16-18,28], although these studies used a different setting, e.g. the study of Butzlaff et al. passed out the questionnaires to the GPs during peer educational outreach visits [16]. Our findings are also supported by Brown and colleagues: in their survey, journal articles, as well as local grand rounds and regional meetings (authors note: comparable to QCs) were the most preferred CME-media [29]. The low appraisal of meetings with pharmaceutical representatives could be an effect of social desirability – it is a topic of current discussion in both national and international medical journals [30,31]. However, another survey of a representative sample of GPs in Germany found a higher frequency of contacts with pharmaceutical representatives [18]. Despite these limitations our results should be taken into consideration when planning learning activities for practicing GPs, because CME/CPD programs could be more effective if they used the preferred learning media.

### Internet, e-learning, and CME

Recently published studies demonstrated that e-learning could have a major impact on knowledge transfer and changes in learning behavior [9,32-34]. Other authors were very optimistic about the use of new technology for CME activities [35,36]. From the GPs' preferences we were able to show that new media does not seem to play an important role in the learning/educational activities of German GPs. Independent of individual learning types, barriers to the use of the internet for CME still exist. Contrary to Wall et al. (2005) we found no significant differences between participation in Internet CME and other demographic or office characteristics. Recent studies suggest that the limited computer literacy of GPs might be responsible for these findings [16,34,35]. However, in our survey nearly 75% of the GPs used the Internet at least once a week, but they did not use it regularly for CME activities. These findings are in accordance with other study findings [18,22,37,38]. There are many potential reasons for this: even though many CME-websites exist, the usability of some sites is not sufficient. The quality of the content is also very heterogeneous [37]. On the other hand GPs seem to be very "traditional" which means that they rarely changed their previously gained learning preferences [17,38-40].

Sargeant and colleagues added two other aspects: The first was the capacity of on-line CME to meet individual learning preferences, which, in turn, was influenced by the quality of the program, the degree of self-pacing or self-direction, opportunity for reflection, and educational design. The second was the quality and quantity of interpersonal interaction, which was shaped by perceptions of social comfort, the educational value of interactions, and the role of the facilitator [41].

Another reason for these results could be the disconnection between online CME developers and the educators, comparable to the misunderstanding between the producers of knowledge and practicing GP suggested by Beaulieu et al. [42,43]. A first step to overcome this gap is to discuss this with both the developers and the users of E-Learning environments [44]. Further – particularly qualitative – studies could be contributed to this process.

### Blended Learning – a smooth transition for usage of new educational media?

Accessing for CME content at any time, in any chosen surrounding and without time schedules represents an attractive option for doctors in clinical practice [45-47]. But e-learning can neither stand alone nor replace conventional ways of learning [13]. Whereas students are more open to adapt modern technologies and environments into their learning activities [11,48], our study suggests that GPs are more likely to continue their common pathways of knowledge acquisition. This is comparable to findings in other countries [38]. Having this in mind one option to implement new learning technologies might include the combination of lectures with matching e-learning sessions or scripts or a blending of all three modalities ("blended learning") [49-53]. Of course for a lot of students WEB 2.0 tools like Blogs, Wikis and Podcasts are part of their daily studies [11,54,55]. So maybe the next generation of GPs will be favorable toward the internet for training and this will then complement face-to-face learning meetings like QCs [56].

## Conclusion

Despite a growing use of the Internet it seems that German GPs favor "classical/traditional" settings for their learning activities. These results should be taken into consideration when planning CME or CPD programs or other learning activities.

## Competing interests

None of the involved persons has a conflict of interests. More particularly, there are no financial or personal relations or dependences to other persons or organizations which might restrict the autonomy of activities. The work was supported by a grant from the Federal Ministry of Education and Research (BMBF) under project number 01GK0512. Any opinions, conclusions and proposals in the text are those of the authors and do not necessarily represent the views of the Ministry.

The study was carried out according to the guidelines of the study manual and the standards of "good epidemiological practice". The WIDA-trial is registered in an international register (to take place under ISRCTN36550981) and has a positive vote of the ethics committee of Witten/Herdecke University (Nr. 42/2006) [19].

## Authors' contributions

HCV conceived and developed this survey and drafted the manuscript. He collected and collated the data and assisted with statistical analysis. MB helped to design the study and contributed to draft the manuscript. MR helped to design the study, assisted in methodological aspects of the survey and contributed to draft the manuscript. TO also assisted in the survey design, performed the statistical analysis, and drafted the manuscript. All authors read and approved the final manuscript.

## Pre-publication history

The pre-publication history for this paper can be accessed here:



## References

[B1] Batalden PB, Mohr JJ (1997). Building knowledge of health care as a system. Qual Manag Health Care.

[B2] Bero LA, Grilli R, Grimshaw JM, Harvey E, Oxman AD, Thomson MA (1998). Closing the gap between research and practice: an overview of systematic reviews of interventions to promote the implementation of research findings. The Cochrane Effective Practice and Organization of Care Review Group. Bmj.

[B3] Davis D, Haynes RB, Chambers L, Neufield VR, McKibbon A, Tugwell P (1984). The Impact of CME. A Methodological Review of the Continuing Medical Education Literature. Eval Health Prof.

[B4] Davis D, O'Brien MA, Freemantle N, Wolf FM, Mazmanian P, Taylor-Vaisey A (1999). Impact of formal continuing medical education: do conferences, workshops, rounds, and other traditional continuing education activities change physician behavior or health care outcomes?. Jama.

[B5] Beyer M, Gerlach FM, Flies U, Grol R, Krol Z, Munck A, Olesen F, O'Riordan M, Seuntjens L, Szecsenyi J (2003). The development of quality circles/peer review groups as a method of quality improvement in Europe. Results of a survey in 26 European countries. Fam Pract.

[B6] Bloom BS (2005). Effects of continuing medical education on improving physician clinical care and patient health: a review of systematic reviews. Int J Technol Assess Health Care.

[B7] Burrows P (2003). Continuing professional development: filling the gap between learning needs and learning experience. Education for Primary Care.

[B8] Davis D, Evans M, Jadad A, Perrier L, Rath D, Ryan D, Sibbald G, Straus S, Rappolt S, Wowk M (2003). The case for knowledge translation: shortening the journey from evidence to effect. Bmj.

[B9] Wall TC, Mian MA, Ray MN, Casebeer L, Collins BC, Kiefe CI, Weissmann N, Allison JJ (2005). Improving Physician Performance Through Internet-Based Interventions: Who Will Participate?. J Med Internet Res.

[B10] Casebeer L, Bennett N, Kristofco R, Carillo A, Centor R (2002). Physician Internet medical information seeking and on-line continuing education use patterns. J Contin Educ Health Prof.

[B11] Boulos MN, Maramba I, Wheeler S (2006). Wikis, blogs and podcasts: a new generation of Web-based tools for virtual collaborative clinical practice and education. BMC Med Educ.

[B12] Cook DA (2005). The research we still are not doing: an agenda for the study of computer-based learning. Acad Med.

[B13] Mamary EM, Charles P (2000). On-site to on-line: barriers to the use of computers for continuing education. J Contin Educ Health Prof.

[B14] Verhoeven AA, Boerma EJ, Meyboom-de Jong B (1995). Use of information sources by family physicians: a literature survey. Bull Med Libr Assoc.

[B15] Haug JD (1997). Physicians' preferences for information sources: a meta-analytic study. Bull Med Libr Assoc.

[B16] Butzlaff M, Koneczny N, Floer B, Vollmar HC, Lange S, Kunstmann W, Köck C (2002). [Primary Care Physicians, Internet and New Knowledge. Utilization and Efficiency of New Educational Media]. Med Klin (Munich).

[B17] Vollmar HC, Ostermann T, Hinz A, Rieger MA, Butzlaff ME (2008). [Primary care physicians, internet and educational media. Preferences, usages and appraisal in a 6-year comparison]. Med Klin (Munich).

[B18] (2007). Internet für Ärzte. Wie niedergelassene Ärzte (APIs) medizinische Seiten im Internet nutzen. Eine Umfrage im Rahmen der LA-MED API-Studie. http://www.la-MED.de.

[B19] Vollmar HC, Butzlaff ME, Lefering R, Rieger MA (2007). Knowledge translation on dementia: a cluster randomized trial to compare a blended learning approach with a "classical" advanced training in GP quality circles. BMC Health Serv Res.

[B20] Cronbach L (2006). Coefficient alpha and the internal structure of tests. Psychometrika.

[B21] Wolf FM, Gruppen LD, van Voorhees C, Stross JK (1986). Dimensions of Motivation for Continuing Medical Education of Primary Care Physicians. Eval Health Prof.

[B22] Blakely JT, Sinkowitz-Cochran RL, Jarvis WR (2006). Infectious diseases physicians' preferences for continuing medical education on antimicrobial resistance and other general topics. Infect Control Hosp Epidemiol.

[B23] Newby TJ, Alter PA (1989). Task Motivation: Learner Selection of Intrinsic versus Extrinsic Orientation. Educational Technology Research and Development.

[B24] Sansone C, Harackiewicz J, Eds (2000). Extrinsic and Intrinsic Motivation The Search for Optimal Motivation and Performance.

[B25] Beal DJ, Dawson JF (2007). On the Use of Likert-Type Scales in Multilevel Data. Organizational Research Methods.

[B26] Grunddaten zur Vertragsärztlichen Versorgung in Deutschland. http://www.kbv.de/publikationen/125.html.

[B27] Edwards P, Roberts I, Clarke M, DiGuiseppi C, Pratap S, Wentz R, Kwan I (2002). Increasing response rates to postal questionnaires: systematic review. Bmj.

[B28] Kunstmann W, Butzlaff M, Böcken J, Braun B, Schnee M (2004). Ärztliche Therapiefreiheit und Fortbildungspflicht – ein Widerspruch? Perspektiven und Einschätzungen aus der Ärzteschaft. Gesundheitsmonitor 2004 Die ambulante Versorgung aus Sicht von Bevölkerung und Ärzteschaft.

[B29] Brown TT, Proctor SE, Sinkowitz-Cochran RL, Smith TL, Jarvis WR (2001). Physician preferences for continuing medical education with a focus on the topic of antimicrobial resistance: Society for Healthcare Epidemiology of America. Infect Control Hosp Epidemiol.

[B30] Korzilius H, Rieser S (2007). Pharmaberater. Für mache Fachmann, für andere Buhmann. Deutsches Ärzteblatt.

[B31] Mueller PS, Hook CC, Litin SC (2007). Physician preferences and attitudes regarding industry support of CME programs. Am J Med.

[B32] Chumley-Jones HS, Dobbie A, Alford CL (2002). Web-based learning: sound educational method or hype? A review of the evaluation literature. Acad Med.

[B33] Fordis M, King JE, Ballantyne CM, Jones PH, Schneider KH, Spann SJ, Greenberg SB, Greisinger AJ (2005). Comparison of the instructional efficacy of Internet-based CME with live interactive CME workshops: a randomized controlled trial. Jama.

[B34] Cobb SC (2004). Internet continuing education for health care professionals: an integrative review. J Contin Educ Health Prof.

[B35] Kripilani S, Cooper HP, Weinberg AD, Laufman L (1997). Computer-Assisted Self-Directed Learning: The Future of Continuing Medical Education. Journal of Continuing Education in the Health Professions.

[B36] Alguire PC (2004). The future of continuing medical education. Am J Med.

[B37] Vollmar HC, Schurer-Maly CC, Frahne J, Lelgemann M, Butzlaff M (2006). An e-learning platform for guideline implementation–evidence- and case-based knowledge translation via the Internet. Methods Inf Med.

[B38] Verhoeven AAH (1999). Information-Seeking by General Practitioners.

[B39] Bower EA, Girard DE, Wessel K, Becker TM, Choi D (2008). Barriers to innovation in continuing medical education. J Contin Educ Health Prof.

[B40] Ruf D, Berner MM, Kriston L, Maier I, Harter M (2008). [General practitioners online: the conditions are good, but use of the Internet for continuing medical education found to be poor]. Z Evid Fortbild Qual Gesundhwes.

[B41] Sargeant J, Curran V, Jarvis-Selinger S, Ferrier S, Allen M, Kirby F, Ho K (2004). Interactive on-line continuing medical education: physicians' perceptions and experiences. J Contin Educ Health Prof.

[B42] Berman NB, Fall LH, Maloney CG, Levine DA (2006). Computer-Assisted Instruction in Clinical Education: a Roadmap to Increasing CAI Implementation. Adv Health Sci Educ Theory Pract.

[B43] Beaulieu M-D, Proulx M, Jobin G, Kugler M, Gossard F, Denis J-L, Larouche D (2008). When is Knowlege Ripe for Primary Care?: An Exploratory Study on the Meaning of Evidence. Eval Health Prof.

[B44] Vollmar HC, Waldmann U-M, Sönnichsen A, Schürer-Maly CC, Gensichen J (2007). Perspektiven von E-Learning in der Allgemeinmedizin – eine Delphi-Studie unter Berücksichtung von Experten und Interessenten. Zeitschrift für Allgemeinmedizin.

[B45] Schultze-Mosgau S, Zielinski T, Lochner J (2004). Web-based, virtual course units as a didactic concept for medical teaching. Med Teach.

[B46] McKimm J, Jollie C, Cantillon P (2003). ABC of learning and teaching: Web based learning. Bmj.

[B47] Cook DA, Dupras DM, Thompson WG, Pankratz VS (2005). Web-based learning in residents' continuity clinics: a randomized, controlled trial. Acad Med.

[B48] Ruiz J, Mintzer M, Leipzig R (2006). The Impact of E-Learning in Medical Education. Academic Medicine.

[B49] Gordon DL, Issenberg SB, Gordon MS, Lacombe D, McGaghie WC, Petrusa ER (2005). Stroke training of prehospital providers: an example of simulation-enhanced blended learning and evaluation. Med Teach.

[B50] King RV, Murphy-Cullen CL, Mayo HG, Marcee AK, Schneider GW (2007). Use of computers and the Internet by residents in US family medicine programmes. Med Inform Internet Med.

[B51] Gold JP, Begg WB, Fullerton D, Mathisen D, Olinger G, Orringer M, Verrier E (2004). Successful implementation of a novel internet hybrid surgery curriculum: the early phase outcome of thoracic surgery prerequisite curriculum e-learning project. Ann Surg.

[B52] Karnath BM, Das Carlo M, Holden MD (2004). A comparison of faculty-led small group learning in combination with computer-based instruction versus computer-based instruction alone on identifying simulated pulmonary sounds. Teach Learn Med.

[B53] Shaffer K, Small JE (2004). Blended learning in medical education: use of an integrated approach with web-based small group modules and didactic instruction for teaching radiologic anatomy. Acad Radiol.

[B54] Taradi SK, Taradi M, Radic K, Pokrajac N (2005). Blending problem-based learning with Web technology positively impacts student learning outcomes in acid-base physiology. Adv Physiol Educ.

[B55] Valcke M, De Wever B (2006). Information and communication technologies in higher education: evidence-based practices in medical education. Med Teach.

[B56] Wiecha J, Barrie N (2002). Collaborative online learning: a new approach to distance CME. Acad Med.

[B57] Slotnick HB, Raszkowski RR, Jensen CE, Wentz DK, Christman Kuntz TA (1994). Physician preferences in CME including insights into education versus promotion. Journal of Continuing Education in the Health Professions.

[B58] Hayward RS, Guyatt GH, Moore KA, McKibbon KA, Carter AO (1997). Canadian physicians' attitudes about and preferences regarding clinical practice guidelines. Cmaj.

[B59] Tinsley JA, Shadid GE, Li H, Offord KP, Agerter DC (1998). A survey of family physicians and psychiatrists. Psychotropic prescribing practices and educational needs. Gen Hosp Psychiatry.

[B60] Smith TL, Sinkowitz-Cochran RL, Jarvis WR (2000). Physician preferences for educational media. Infect Control Hosp Epidemiol.

[B61] Butzlaff M, Telzerow A, Lange S, Kruger N (2001). [Physicians, internet and new knowledge. Utilization and efficiency of new continuing education media in the hospital] Ärzte, Internet und neues Wissen. Nutzung und Effizienz von neuen Weiterbildungsmedien im Krankenhaus. Med Klin (Munich).

[B62] Slotnick HB, Harris TR, Antonenko DR (2001). Changes in learning-resource use across physicians' learning episodes. Bull Med Libr Assoc.

[B63] Stancic N, Mullen PD, Prokhorov AV, Frankowski RF, McAlister AL (2003). Continuing medical education: What delivery format do physicians prefer?. Journal of Continuing Education in the Health Professions.

